# Microstructural, Mechanical and Radiological Characterization of Mortars Made with Granite Sand

**DOI:** 10.3390/ma14195656

**Published:** 2021-09-28

**Authors:** Francisca Puertas, José Antonio Suárez-Navarro, Alfredo Gil-Maroto, Ana María Moreno de los Reyes, Catalina Gascó, Alicia Pachón, María del Mar Alonso

**Affiliations:** 1 Department of Materials, Eduardo Torroja Institute for Construction Sciences (IETcc-CSIC), 28033 Madrid, Spain; agil@ietcc.csic.es (A.G.-M.); ana.moreno@ietcc.csic.es (A.M.M.d.l.R.); apachon@ietcc.csic.es (A.P.); mmalonso@ietcc.csic.es (M.d.M.A.); 2 Department of Environment, Environmental Radioactivity and Radiological Surveillance (CIEMAT), Avenida Complutense 40, 28040 Madrid, Spain; ja.suarez@ciemat.es (J.A.S.-N.); ctlngasleon@outlook.es (C.G.)

**Keywords:** mortars, granite sands, eco-efficient cements, natural radioactivity, microstructure, gamma spectrometry

## Abstract

The study reported the effect of granite sand on strength and microstructural developments in mortars prepare from OPC with a high coal fly ash (FA) content or from hybrid alkaline cements. The radiological behaviour of the resulting mortars was compared to materials prepared with siliceous sand (with particles sizes of <2 mm) and the relationship between such radiological findings and mortar microstructure and strength was explored. A new method for determining natural radionuclides and their activity concentration Index (ACI) on cement mortars (specifically to solid 5-cm cubic specimens) was applied and validated. The microstructural changes associated in mortars have no effect on mortar radiological content measurements. The mortars with granite sand exhibited very high ACI > 0.96, which would ultimately limit their use. A conclusion of interest is that where information is at hand on the starting materials (OPC, FA, sand, admixtures), their proportions in the mortar and the mixing liquid content (water or alkaline activators) their radiological content is accurately predicted. The inference is that a mortar’s radiological content and ACI can be known prior to mixing, providing a criterion for determining its viability. That in turn lowers environmental risks and the health hazards for people in contact with such materials.

## 1. Introduction

Granite stone has been used as a construction material since antiquity, primarily for ornamental purposes in aqueducts, buildings and other civil works [1]. It is still used today in countertops, walkways, staircases and footbridges, where it is appreciated for its high wearing resistance and low absorption and permeability [2]. Albeit more incidentally, it may also be used as an aggregate or filler in cement mortars and concretes in the absence of other alternatives or when the available natural aggregate is of very low quality (liable to induce the alkali-aggregate reaction, for instance).

Granite cutting generates massive amounts of waste in solid form (during quarrying) and sludge (during processing) [3]. Managing vast quantities of granite sludge can be a substantial challenge, for the waste must be kept under conditions safe both for the surrounding environment and its human population. In addition, transporting the sludge to and depositing it in a spoil heap carry no small cost. One solution to those environmental and economic problems is to recycle this waste in other industrial processes, which would also afford new business opportunities. Some studies have shown granite sludge to have substantial potential as a raw material for the ceramic industry [4,5,6,7]. The use of steel or other abrasive material blades to cut the granite generates sludge with a high iron oxide content, particularly suitable for manufacturing stained ceramics [8].

As noted earlier, although using crushed granite as an aggregate or filler in mortars and concretes is not standard practice, partially replacing cement and/or fines (sand) in mortars and concretes with the sludge and waste from hewing operations is environmentally beneficial and contributes to sustainability. A number of authors [3,9,10,11,12] have studied the effect of including such granite waste as a replacement for cement or fine aggregate on the strength and durability of the respective mortars and concretes. Felixhala et al. [12] observed that substituting granite sludge for part of the natural sand in concrete enhanced the material’s mechanical strength without altering its soundness or fresh state behaviour. In a similar vein, Ramos et al. [10] showed that replacing up to 10 wt% cement with fine-grain granite waste furthered the formation of a denser matrix, lowered alkali-silica reaction (ASR)-induced expansion by 38% and raised chloride resistance by nearly 70% without affecting mortar workability or strength. The beneficial effects of replacing fines (sand) with granite materials on mortar and concrete strength and durability would therefore appear to have been proven. Hence, the importance of further exploring granite materials as components in mortars and concretes, in light of the environmental benefits and enhanced sustainability to be reaped.

However, the high activity concentrations of the natural uranium, thorium, actinium and potassium series in granite rock, where the (Th)/(U) mass ratio ranges from 2.25 to 4.67, must not be overlooked [13,14]. Such high U and Th mass concentrations are a direct consequence of granite formation, for when Earth crust rocks fuse, U and Th remain in a liquid phase and are subsequently taken up into compounds with a high SiO_2_ concentration [15]. Granite is deemed to be the primary source of the uranium mobilised in the environment. The U (IV) found in the stone is oxidised by external agents to U (VI) such as UO_2_^2+^ that forms very stable soluble complexes with the carbonates, sulphates, chlorides and phosphates present in ground water [16,17]. Ra and Th may also be mobilised, although to a lesser extent than U [18].

Very recent studies [19,20,21] have shown that granite particle size and mineralogical composition are related to its natural radionuclide content. The activity concentrations of the thorium, uranium and actinium natural decay series in granite have been observed to be higher in the finest fraction (<2 mm). A correlation has likewise been identified between Th content and the proportion of MgO and Fe_2_O_3_, minerals normally present in granite micas. Those same studies confirmed that including granite as aggregate in mortars raised the activity concentration (ACI) in those materials [22].

The inference of the foregoing is that: a) granite can be included in mortars and concretes as sludge or fine solid waste; and b) such granite fines are the primary source of the natural radionuclides in the end products (mortars and concretes). That in turn generates a need for a fuller understanding of the effect of these granite fines when included as sand in mortars of varying nature and composition, as well as the effect of their pore microstructure on total radiological content. That matter, essential to determine activity concentration more accurately in these construction materials [23,24], has been scantly explored to date.

The present authors recently established a new way to measure the radiological content in Portland cement pastes with and without supplementary cementitious materials (SCMs) [25,26]. That new approach is based on gamma spectrometric analysis of solid monolithic (specifically 5-cm cubic) specimens rather than on the powdered or ground samples conventionally used.

As noted, recycling granite waste as aggregate in mortars and concretes is both environmentally beneficial and contributes to the industry’s sustainability. The latter can be enhanced if in addition to recycled aggregate, eco-efficient cements are used to prepare the respective mortars. With their small carbon footprint and low energy consumption, Portland cements with a high SCM content and alkali-activated cements or geopolymers are among the eco-efficient binders most widely deployed [27,28,29]. Of particular interest in this regard are the so-called hybrid cements, which may bear up to 70% geopolymer and just 30% OPC.

The study reported here ascertained the effect of granite sand on strength development in mortars prepared from OPC with a high coal fly ash content or from hybrid alkaline cements. The radiological behaviour of the resulting mortars was compared to materials prepared with siliceous sand (with particles sizes of <2 mm) and the relationship between such radiological findings and mortar microstructure and strength was explored. A new method for determining natural radionuclides and their activity concentration (ACI) values in unground solids was applied for the first time to cement mortars, and more specifically to solid 5-cm cubic specimens.

## 2. Materials and Methods

### 2.1. Materials

Three types of materials were used in this study. The binders included a CEM I 52.5R Portland cement (OPC) and an ASTM type F fly ash (FA). One of the two sands was a European standard EN-196-1:2018-compliant siliceous aggregate (S) and the other granite fines (G). The third component was a polycarboxylate superplasticiser (SP).

The chemical composition of the binders and sands used, found on a Bruker S8 XRF spectrometer (Bruker, Billerica, MA, USA), is given in Table 1. The vitreous phase content in FA was determined by a selective 1% HF attack to be 62.09% [30].

Quantitative and qualitative mineralogical composition for OPC and FA were recorded with a Bruker AXL Advance D8 diffractometer fitted with an ultrafast Lynxeye X-ray detector (Bruker, Billerica, MA, USA) and a 2.2 kW copper anode, configured for use without a monochromator. The mineralogical phases detected were quantified with Rietveld analysis using DIFFRAC-EVA.V4.2 software and the Crystallography Open Database (COD). The relative quantitative XRD findings for the two materials are given in Table 2 [26].

The particle size distribution of both materials, determined by laser diffraction on a Mastersizer analyser fitted with a He-Ne 632.8 nm laser, is given in Table 3. The Blaine specific surface of the two starting materials was determined further to European standard EN 196-6:2010 [32].

The likewise XRD-determined quantitative mineral composition of the sands, in turn, is listed in Table 4.

The particle size distribution used was the same in the siliceous and granite sands to rule out the possible effect of that parameter on the findings, for the aim was to compare their microstructure under identical conditions. The granite sand was consequently sieved to the same particle size as the EN-196-1:2018-standardised product [33]. Further to the particle size distribution of the two sands given in Table 5 all the particles in both were under 2 mm. The two aggregates exhibited similar densities at around 2.6 g/mL. Water absorption in the siliceous material was 0.19% and in the granite 0.15%, measured in both cases as set out in EN-1097-6:2001 [34].

Sika’s ViscoCrete HC-20 was the superplasticiser (SP) added to the mortars.

### 2.2. Mortar Preparation

All the mortars were prepared to a sand/binder ratio of 3/1. The mixing water content in all the mortars was as required to ensure the 105 ± 5 cm slump recommended in Spanish and European standard UNE-EN-1015-3 [35].

The parameter values defined for mortar preparation were as follows.

(a) Binder type:
  - Cement with high FA content: 70% OPC + 30% FA
  - Hybrid alkaline cement: 30% OPC + 70% FA
(b) Sand type:
  - Siliceous (S)
  - Granite (G)
(c) Presence/absence of the superplasticiser (SP). SP (1 wt% of the binder) was added only to the 70% OPC + 30% FA mortars, for the conventional superplasticisers used in Portland cement concrete are known to be ineffective in alkaline-activated or geopolymer systems.

**Figure 1:**
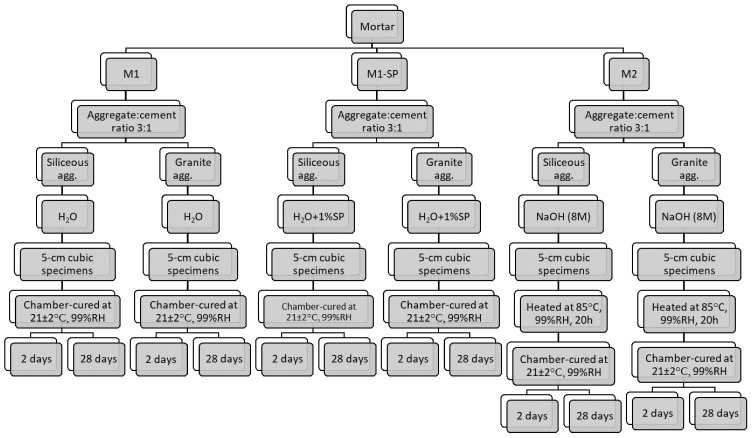
Mortar preparation flow chart.

Table 6 lists all the mortars prepared. The process is outlined in the flowchart in Figure 1. The 70% OPC + 30% FA binders (some of which with SP) were mixed with water, whilst the hybrid cement mortars (30% OPC + 70% FA) were mixed with an 8 M NaOH solution. The curing conditions also varied: the former in a humidity chamber at 20 ± 2 °C and 99% RH and the latter at 85 ± 2 °C and 99% RH for the first 20 h and subsequently through testing age under the same conditions as the OPC + FA materials.

The components specified in Table 6 were used to prepare 5-cm cubic specimens as described in European standard EN 196-1:2018 [33]. The specimens were stored in a humidity chamber (21 ± 2 °C and 99% RH) until tested, i.e., after 2 days or 28 days curing.

### 2.3. Mechanical and Microstructural Characterisation

The tests conducted on the 5-cm cubic specimens are listed below.

Compressive strength was determined by an Ibertest Autotest-200/10-SW, test frame as described in standard ASTM 109 [36]. The value given is the mean for three specimens per mortar and curing age (2 days or 28 days).

Porosity, in terms of total porosity and pore size distribution, was tested on a Micromeritics Autopore IV 9500 analyser (Micromeritics. Norcross, GA, USA) designed for pressures of up to 32,000 psi, equivalent to a pore size of 0.0067 µm.

Water sorptivity and density were established as per European standard EN 12390-7:2009 [37].

### 2.4. Radiological Characterisation. Statistical Analysis

The gamma spectrometry laboratory where the specimens were analysed is an institution accredited by the Spanish National Accreditation Agency to Spanish, European and international standard UNE-EN ISO/EC 17025:2005 [38]. Three types of HPGe detectors were used to measure the samples: (i) extended range coaxial, (ii) reverse electrode coaxial; and (iii) broad energy. The detectors were fitted with 15 cm thick iron and lead passive shielding. Detector resolution was ≤2.0 keV for the ^60^Co 1.33 MeV photo peak in all cases. Each detector was connected to an electronic chain comprising one amplifier, one analogue-digital converter, one high voltage power supply and a communications antenna interface module (AIM) (all from Canberra Industries). The spectra were logged and subsequently analysed with Genie 2000 software (Canberra Industries, Meriden, CT, USA) [39].

Three types of samples were analysed: (i) anhydrous (cements and siliceous and granite sand) and samples of each liquid used to prepare the cement mortars with the two types of sand, in individual 76 mm diameter, 30 mm high cylindrical polypropylene containers; (ii) 5-cm cubic cement mortar specimens; and (iii) ground cement mortars packed in the containers described in (i). Duplicates of all samples were prepared and the cubes were measured on two of their six sides. The samples were sealed to prevent ^222^Rn emanation and ensure attainment of secular equilibrium with its radioactive daughters. The cubic specimens were coated with a 1 mm layer of epoxy resin and the cylindrical containers were sealed with parafilm and adhesive tape. Detector efficiency was established using LabSOCS software and the procedure described in [25]. The naturally radioactive series radionuclides analysed included [40]: ^234^Th (63.30 (2) keV), ^226^Ra (186.211 (13) keV), ^214^Pb (351.932 (2) keV), ^214^Bi (609.312 (7) keV; 1120.287 (10) keV; 1764.494 (14) keV), ^210^Pb (46.539 (1) keV, ^212^Pb (238.632 (2) keV), ^208^Tl (583.187 (2) keV, ^228^Ac (911.196 (6) keV), ^235^U (163.356 (3) keV; 205.16 (4) keV; 143.767 (3) keV) and ^40^K (1460.822 keV).

The 186.211 KeV photo peak was corrected for ^235^U interference to find ^226^Ra activity concentration (and the 1460.822 photopeak for ^228^Ac to find ^40^K activity). In both cases correction was based on the algorithm proposed in [41].

Equation (1) [42,43] was applied to find end construction product, i.e., OPC + FA and hybrid cement cube, activity concentration (*ACI*):

(1) ACI=AR 226a300+AT 232h200+AK 403000

where AR 226a is activity concentration for ^226^Ra (determined from the ^226^Ra 186.211 keV photopeak); AT 232h is activity concentration for ^232^Th (determined from ^212^Pb); and AK 40 is ^40^K activity concentration, all expressed in Bq kg^−1^. An ACI value greater than 1 would mean an excess of effective dose greater than 1 mSv per year, which corresponds to the allowed dose for the general public [44].

The final activity concentrations were determined from the weighted mean of the individual values (Equation (2)). The uncertainty associated with activity concentration was calculated in keeping with the Bambyneck [45] criterion, i.e., as the higher of the values calculated from Equations (3) and (4).

(2) $$A=\frac{\sum_{i=1}^{N}\left(A_{i}*u\left(A_{i}\right)\right)}{u\left(A_{i}\right)}$$

(3) $$u{\left(A\right)}_{ext}=\sqrt{\frac{\sum_{i=1}^{N}u{\left(A\right)}_{i}\cdot {\left(A_{i}-A\right)}^{2}}{\frac{N}{N-1}\cdot \sum_{i=1}^{N}u{\left(A\right)}_{i}}}$$

(4) $$u{\left(A\right)}_{int}=\sqrt{{\left(\sum_{i=1}^{N}u{\left(A\right)}_{i}^{-2}\right)}^{-1}}$$

where *A_i_* is the *i*-th activity concentration and *u*(*A_i_*) is the uncertainty associated with that *i*-th concentration.

The activity concentrations for the ground cement mortars were compared to the values calculated using the values of each individual component with one-way ANOVA (analysis of variance), defining statistical significance at α = 0.05. The possible effects of curing time and cement mortar composition for both the siliceous and granite sand materials were compared with two-way ANOVA and statistical significance α = 0.05 The null hypothesis (H_0_) for both ANOVAs was the absence of significant differences in the mean values for the datasets studied. H_0_ was accepted for probabilities of *p* > 0.05 [46,47].

## 3. Results and Discussion

The findings for mechanical strength and microstructure of the mortars prepared with eco-efficient, high FA content or hybrid alkaline cements and granite or siliceous sand are discussed below. The effect of changes in the pore network induced with the addition of a superplasticiser and concomitant reduction of mixing water is described. The item on the results of the radiological analyses addresses the effect of the use of granite sand on mortars in terms of their naturally occurring radionuclide content, along with the relationship observed between radiological content and mortar microstructure. Lastly, the applicability of the new methodology for measuring gamma spectrometry in solid (unground) samples, validated for cements with and without SCMs, is explained.

### 3.1. Mechanical and Microstructural Characterisation

**Figure 2:**
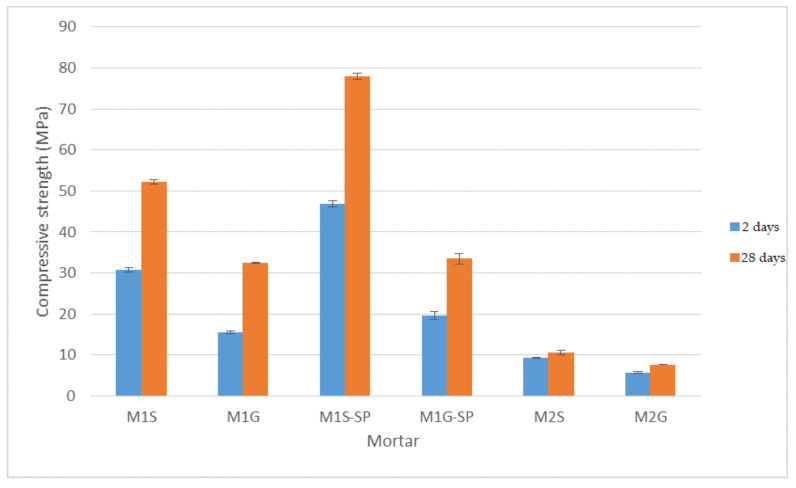
2-day and 28 days mortar compressive strength.

The compressive strength of the mortars prepared and tested is showed in Figure 2. The materials bearing siliceous sand, M1S and M2S, were observed to have higher strength than their granite sand-bearing counterparts, M1G and M2G, in the 2 days and 28 days mortars made with both FA-additioned and hybrid alkaline cement. These findings were consistent with the liquid content required to ensure the same slump in all mortars (Table 6). The greater amount of mixing liquid required in the mortars with granite than in those with siliceous sand translated into higher water absorption and total porosity (see Table 7 and Table 8) and consequently lower strength. Those effects were attributable to the irregular shape and rougher surface texture of the granite fines. Earlier authors reporting similar results [9,10,11] showed that high granite sludge or fines contents in mortar and concretes lowered mechanical strength and had an adverse effect on fresh state performance.

The SP impacted siliceous (M1S-SP) and granite (MG1-SP) sand mortars differently. In the former the L/S ratio dropped by 30% (Table 6), compared to a decline of just 18% in the latter. The explanation for that differential behaviour is again to be found in the surface characteristics of the granite sand. Since the SP prompted a lesser reduction of the L/S ratio in granite than in siliceous sand mortars, its inclusion induced a smaller rise in strength in the former.

Binder composition also affected mortar mechanical performance. The ones additioned with FA (M1S and M1G) had higher compressive strength than the hybrid cement mortars (M2S and M2G) at both ages studied. Another difference was that strength rose between 2 days and 28 days in the FA-bearing but not in the hybrid materials (Figure 2). The much higher L/S ratio required by hybrid mortars than by mortars M1S and M1G (Table 6) was associated with their composition (higher FA content; greater mixing liquid alkalinity). In addition, in these geopolymers initial curing time is known to favour ash dissolution and reactivity, generating three-dimensional reaction products [29], and that such thermal curing inhibits the development of mechanical strength over time, contrary to the behaviour observed in FA-additioned OPC mortars. Hence, the absence of any substantial change in compressive strength between the 2 d and 28 d geopolymer mortars.

The 2 days and 28 days findings for water absorption, density and total porosity in these mortars are given in Table 7 and Table 8, respectively. The data show that given the same conditions, water absorption and total porosity were lower in siliceous than in granite sand mortars. The findings for all three parameters were wholly consistent with the strength values described above. The hybrid cement mortars (whether with siliceous or granite sand) exhibited the highest water absorption and lowest density.

**Figure 3:**
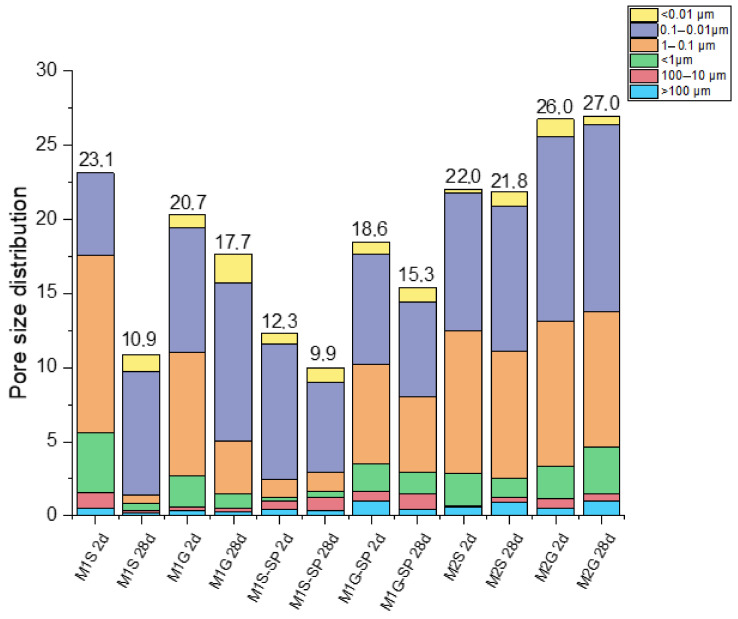
Mortar pore size distribution.

Mortar pore size distribution is depicted in the bar graph in Figure 3. The most visible effect was the decline in total porosity and rise in the percentage of 0.1 µm to 0.01 µm pores with hydration time (2 days to 28 days) in mortars M1S and M1G (with and without SP). In other words, time refined the pore structure. The characteristics of the sands discussed earlier explain the differences in the values observed. No relevant changes are observed on pore size distribution on M2S and M2G mortars with curing time. That is due to the high reaction/activation process produced at first time by effect of curing temperature (85 °C during the first 20 h).

As an analysis of the main results obtained in the mechanical and microstructural characterization is the use of granite sand in mortars with OPC cements with high content of FA and hybrid alkaline binder, is those mortars need more mixing water than those prepared with siliceous sand. That is attributable to the more irregular shape and more porous texture of granite sand, which consequently retains more water. The addition of superplasticisers fluidises FA-bearing OPC mortars prepared with granite sand less than mortars with siliceous sand. The nature of eco-efficient cements used in this study also has a relevant effect in the final microstructure.

### 3.2. Radiological Characterisation

The activity concentrations of the radionuclides present in the individual components used in this study are listed in Table 9. The fly ash (FA) and OPC exhibited the same activity concentrations as reported in [26]. Those values were consistent with the results of earlier studies [19], bearing in mind the particle size distribution of the sands (Table 5). Concentration in the 8 M NaOH solution and the superplasticiser (SP) used to prepare the mortars was found to be below the minimum detectable activity (MDA) threshold.

The activity concentrations recorded for the siliceous sand-bearing mortars prepared with both types of eco-efficient cements in the solid cubes and in the powders obtained by grinding the specimens, as well as the values calculated from the proportions and data in Table 9, are given in Table 10, and the analogous data for the granite sand specimens in Table 11. Activity concentrations were statistically indistinguishable for the solid cube and powder obtained after grinding for both siliceous and granitic aggregate. Firstly, the one-way ANOVA conducted for each sample verified the absence of significant differences among the three means, for the probabilities were consistently *p* > 0.05. That finding attested to the reproducibility of the measurements in all the hardened cement mortar samples, irrespective of the nature of the eco-cement and the sand (siliceous or granite) used in their preparation. Where the proportions of the starting materials (OPC, FA, sand, admixtures) and the composition (water or alkaline activator) and amount of mixing liquid in the mortar were known, the radiological content of the end products could be accurately predicted, yielding values very close to the empirical findings for the hardened and ground mortars. The inference is that a mortar’s radiological content and activity concentration can be known prior to mixing, providing a sound criterion for determining its viability. In other words, only the mortars with a low and controlled radiological content would be prepared, with the concomitant reduction of environmental risks, health hazards for those concerned, preparation time, material and energy consumption and carbon footprint.

ANOVA also verified that none of the binary variables studied (2 days or 28 days curing, nature of eco-efficient cement, absence or presence of superplasticiser) generated significant differences in the activity concentrations recorded, for in all cases the probabilities were greater than 0.05. These findings are extrapolable to the ACI, as this index is determined from the activity concentrations of the gamma emitters present in the final construction material. These results show that there is no correlation between the ACI and the physical properties and microstructural developments. The only effect is the radionuclide concentration of the materials that make up the mortars.

**Figure 4:**
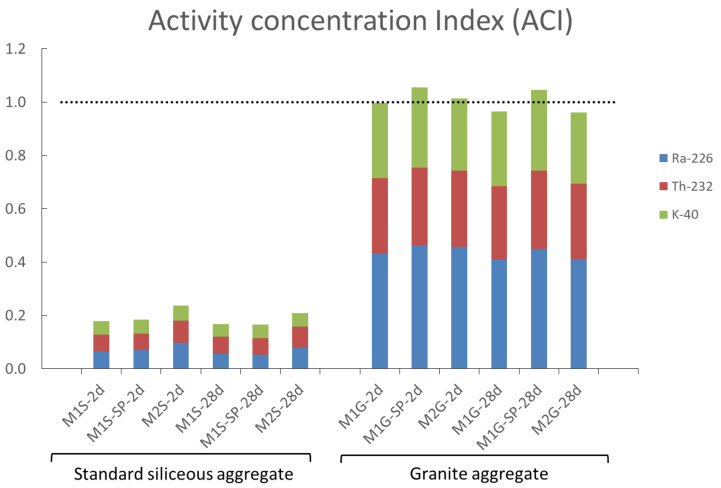
Activity concentration for cubic of OPC-FA and hybrid cement mortars prepared with standard siliceous or granite fines.

The activity concentrations (Equation (2)) for the cubic mortar specimens (with two types of eco-cements) and the two sands used here are graphed in Figure 4. The concentrations found for three of the six mortars bearing granite sand were over 1. The lowest activity in the other three was 0.962. Therefore, the use of granite sand (with particle size <2 mm) in construction materials would be restricted on the grounds of the dose rate. More specifically, constrained usage would be attributable to the uranium series radionuclides and especially ^226^Ra that account for most of the dose. The higher radionuclide concentration observed in the uranium natural decay series was due to the hydrothermal formation of that element, which would also explain the higher values recorded for the U than for the Th series [48].

## 4. Conclusions

The most prominent conclusions to be drawn from the present study are listed below.

The implications of the findings on the effect of granite sand on strength development in eco-efficient cements include the following:

(1) Given the same particle size of siliceous and granite sand, mortars made with the latter call for more mixing water, whether the cement used is an FA-bearing OPC or a hybrid alkaline binder. That is attributable to the more irregular shape and more porous texture of granite sand, which consequently retains more water. The ultimate outcome is lower mechanical strength and higher porosity in the respective mortars.
(2) The addition of superplasticisers fluidises FA-bearing OPC mortars prepared with granite sand less than mortars with siliceous sand. That lesser effect on the mixing water needed is likewise due to the surface characteristics of granite sand.
(3) The inference of the foregoing is that mortars made with both types of eco-efficient cements develop lower mechanical strength and higher porosity when prepared with granite than with siliceous sand.

The implications of the analysis and findings on the effect of granite sand on strength development in eco-efficient cements and the radiological behaviour of the respective mortars include.

(1) The new system for determining natural radionuclides in 5-cm cubic specimens is valid. To date that methodology has been verified in cement pastes, but not in mortars. Validity applies to both OPC + FA and hybrid alkaline cement mortars made with either siliceous or granite sand.
(2) The microstructural changes associated with the use of superplasticisers lower the mixing water content needed, and consequently the porosity and pore size distribution have no effect on mortar radiological content measurement.
(3) Mortars with granite sand have very high activity concentrations, >0.96, whilst several exceed the threshold value of 1. The uranium series radionuclides and especially ^226^Ra, which largely account for the dose rate, would therefore determine restricted use of granite sand. The high radionuclide concentration in the uranium natural decay series is due to the hydrothermal formation of that element, which would also explain the higher values observed for the U than for the Th series.

A conclusion of particular interest is that where information is at hand on the proportions of the starting materials (OPC, FA, sand, admixtures) and the liquid (water or alkaline activator) to solid ratio, mortar radiological content can be accurately predicted and is very close to the empirically determined values for hardened and ground mortars. The inference is that a mortar’s radiological content and activity concentration can be known prior to mixing, providing a sound criterion for determining its viability. That in turn entails lower environmental risks and health hazards for people in contact with those materials, as well as a smaller carbon footprint and savings in preparation time, materials and energy consumption.

## Figures and Tables

**Table 1:** Chemical composition of OPC, FA and sands S and G (% wt.).

Material	CaO	SiO_2_	Al2O_3_	MgO	Fe_2_O_3_	MnO	Na_2_O	P_2_O_5_	SO_3_	K_2_O	TiO_2_	Otros	LoI ^1^	I.R. ^2^
OPC	64.47	20.29	5.67	0.84	2.35	-	0.11	0.14	2.91	0.97	0.24	0.17	2.97	1.07
FA	4.78	42.44	26.95	0.80	18.40	-	0.50	0.20	1.44	1.53	1.07	0.03	1.63	7.78
S	0.15	92.95	4.11	0.15	0.76	0.03	-	-	0.04	1.17	0.09	0.36	0.19	-
G	0.90	71.90	15.39	0.52	1.39	0.03	-	-	0.02	5.56	0.25	3.34	0.70	-

^1^ Loss on ignition, as per EN-196-2: 2014 [31]. ^2^ Insoluble residue, as per EN-196-2:2014 [31].

**Table 2:** OPC and FA quantitative mineralogical composition (wt%).

**Material**	**OPC**							
**Phase**	C_3_S	C_2_S	C_3_A	C_4_AF	Gypsum	Bassanite	Calcite	
(wt%)	64.21	13.16	8.98	5.82	1.77	1.64	4.42	
	**FA**							
**Material Phase**	Amorphous phase	Quartz	Mullite	Hematite	Magnesium ferrite	Magnetite	Maghemite	Calcite
(wt%)	62.09	7.97	20.43	2.41	3.99	1.65	0.82	0.64

**Table 3:** OPC and FA particle size distribution and Blaine specific surface.

Material	Dv10 (µm)	Dv50 (µm)	Dv90 (µm)	Blaine (m^2^ kg^−1^)
OPC	2.34	9.31	27.01	404.68
FA	1.93	16.13	51.54	451.87

**Table 4:** Quantitative mineralogical composition of siliceous (S) and granite (G) sand (wt%).

Sand	Quartz	Alkaline Feldspars	TiO_2_	Phyllosilicates
S	92.3	7.7	--	--
G	64.8	28.2	2.4	4.6

**Table 5:** Particle size distribution of sands used to prepare the mortars.

Siliceous (S) and Granite (G) Sand Particle Size Distribution	
Sieve (mm)	(%)
2	0
1	34.263
0.5	32.079
0.212	14.952
0.125	12.18
0.063	6.015
≤0.063	0.501

**Table 6:** Mortars prepared.

Mortar	% OPC	% FA	Sand	L/S	Liquid	% SP
M1S	70	30	S	0.50	Water	--
M1S-SP	70	30	S	0.35	Water	1
M1G	70	30	G	0.65	Water	--
M1G-SP	70	30	G	0.53	Water	1
M2S	30	70	S	0.76	8 M NaOH	--
M2G	30	70	G	0.91	8 M NaOH	--

**Table 7:** Physical properties of 2 days mortars.

Mortar	Water Absorption (% wt)	Density (g/mL)	% Total Porosity
M1S	17.75	2.11	23.10
M1S-SP	12.50	2.20	12.34
M1-G	21.72	1.97	20.73
M1G-SP	17.05	2.05	18.61
M2S	22.39	2.02	21.96
M2G	24.01	1.93	26.01

**Table 8:** Physical properties of 28 days mortars.

Mortar	Water Absorption (% wt)	Density (g/mL)	% Total Porosity
M1S	15.68	2.15	10.90
M1S-SP	10.42	2.21	9.86
M1-G	20.06	2.00	17.68
M1G-SP	14.47	2.09	15.37
M2S	22.30	2.01	21.83
M2G	23.32	1.92	26.97

**Table 9:** Activity concentrations for radionuclides in the uranium, thorium, and actinium radioactive series and ^40^K for the seven elements analysed in this study.

Sample	Uranium Series					Thorium Series			Actinium Series	^40^K
	^234^Th	^226^Ra	^214^Pb	^214^Pb	^210^Pb	^228^Ac	^212^Pb	^208^Tl	^235^U	
FA	184.5 ± 6.4	164 ± 13	178 ± 10	159.6 ± 2.9	108.0 ± 3.7	59.6 ± 1.6	66.8 ± 3.8	23.30 ± 0.71	7.82 ± 0.68	292.0 ± 9.2
OPC	20.2 ± 1.3	19.0 ± 1.9	18.8 ± 1.1	17.29 ± 0.42	19.1 ± 1.4	20.14 ± 0.64	21.4 ± 1.6	8.26 ± 0.27	<2.4	235.0 ± 7.4
Sand (S)	11.2 ± 2.8	9.4 ± 5.0	7.26 ± 0.66	6.7 ± 1.1	20.4 ± 3.9	5.97 ± 0.44	7.47 ± 0.50	2.92 ± 0.22	<3.5	141 ± 10
Sand (G)	208 ± 15	179 ± 16	202 ± 11	188 ± 12	227 ± 23	59.6 ± 2.2	64.2 ± 3.7	26.8 ± 1.3	7.9 ± 1.0	1066 ± 46
NaOH 8 M	<3.8	<3.8	<0.9	<0.9	<3.5	<1.2	<0.6	<0.3	<2.2	<4.6
SP	<6.5	<4.6	<0.4	<0.3	<6.5	<0.7	<0.3	<0.2	<1.6	<2.4

The uncertainties cited are for a coverage factor k = 2.

**Table 10:** Activity concentrations (Bq kg^−1^) for: (a) 5-cm cubic hardened hybrid cement specimens; (b) ground mortars; and (c) values calculated from the percentage of individual components used to prepare mortars with siliceous sand.

Sample	Type	Uranium Series					Thorium Series			Actinium Series	^40^K
		^234^Th	^226^Ra	^214^Pb	^214^Pb	^210^Pb	^228^Ac	^212^Pb	^208^Tl	^235^U	
M1S-2d	Cubic specimens	25.7 ± 1.9	19.2 ± 3.0	19.0 ± 1.9	17.8 ± 2.0	26.4 ± 2.4	11.8 ± 1.0	12.59 ± 0.70	5.27 ± 0.33	<1.2	154 ± 13
	Ground samples	23.8 ± 2.1	12.7 ± 2.2	18.0 ± 1.7	17.0 ± 1.8	21.1 ± 2.6	11.76 ± 0.50	12.67 ± 0.86	5.30 ± 0.37	<1.8	138.3 ± 5.9
	Calculated	22.9 ± 1.9	20.2 ± 3.5	19.6 ± 3.5	17.80 ± 0.76	23.8 ± 2.6	11.09 ± 0.33	12.76 ± 0.49	4.79 ± 0.16	0.521 ± 0.045	150.0 ± 6.8
M1S-SP-2d	Cubic specimens	26.4 ± 2.5	20.9 ± 5.7	19.1 ± 2.6	17.3 ± 2.3	26.0 ± 3.1	11.8 ± 1.0	12.31 ± 0.90	5.14 ± 0.23	<1.5	158 ± 15
	Ground samples	21.7 ± 2.0	16.7 ± 2.7	17.8 ± 1.8	17.9 ± 2.3	16.6 ± 4.6	11.90 ± 0.49	12.28 ± 0.74	4.67 ± 0.28	<1.8	144.4 ± 5.3
	Calculated	23.6 ± 2.0	20.8 ± 3.6	20.3 ± 3.6	18.36 ± 0.79	24.5 ± 2.7	11.44 ± 0.34	13.17 ± 0.50	4.94 ± 0.16	0.538 ± 0.047	154.8 ± 7.0
M2S-2d	Cubic specimens	43.8 ± 2.2	29.4 ± 3.8	29.8 ± 1.2	27.7 ± 1.2	40.9 ± 3.6	15.51 ± 0.36	16.48 ± 0.69	6.91 ± 0.31	<2.3	171.4 ± 3.6
	Ground samples	37.5 ± 3.2	30.0 ± 3.5	34.6 ± 1.9	32.9 ± 1.2	33.5 ± 3.9	16.25 ± 0.59	17.0 ± 1.0	6.97 ± 0.34	<1.9	156.3 ± 5.4
	Calculated	35.5 ± 2.0	31.2 ± 3.7	31.9 ± 3.7	28.79 ± 0.81	30.4 ± 2.5	13.80 ± 0.37	15.93 ± 0.65	5.79 ± 0.17	1.15 ± 0.10	146.6 ± 6.5
M1S-28d	Cubic specimens	25.9 ± 2.4	16.8 ± 3.9	20.24 ± 0.81	18.75 ± 0.59	26.3 ± 3.2	12.26 ± 0.79	12.91 ± 0.55	5.61 ± 0.22	<1.6	140.9 ± 6.4
	Ground samples	21.5 ± 1.8	16.2 ± 2.4	18.7 ± 1.9	17.2 ± 2.1	25.3 ± 3.0	12.60 ± 0.61	12.91 ± 0.77	5.20 ± 0.27	<1.7	136.1 ± 4.7
	Calculated	22.9 ± 1.9	20.2 ± 3.5	19.6 ± 3.5	17.80 ± 0.76	23.8 ± 2.6	11.09 ± 0.33	12.77 ± 0.49	4.79 ± 0.16	0.522 ± 0.045	150.0 ± 6.8
M1S-SP-28d	Cubic specimens	27.4 ± 1.9	15.8 ± 5.1	18.6 ± 2.2	17.2 ± 2.1	25.6 ± 4.5	11.6 ± 1.1	12.43 ± 0.71	5.12 ± 0.33	<1.2	153 ± 18
	Ground samples	22.0 ± 2.0	19.4 ± 3.3	21.1 ± 2.7	20.4 ± 3.1	22.6 ± 2.6	12.43 ± 0.92	12.84 ± 0.74	4.91 ± 0.25	<1.3	143.3 ± 8.3
	Calculated	21.8 ± 2.0	19.1 ± 3.6	18.5 ± 3.6	16.77 ± 0.78	22.8 ± 2.7	9.59 ± 0.33	11.20 ± 0.45	4.18 ± 0.16	0.538 ± 0.047	133.2 ± 6.9
M2S-28d	Cubic specimens	41.8 ± 2.9	23.7 ± 1.9	29.0 ± 3.9	26.6 ± 3.7	38.0 ± 3.9	14.3 ± 1.0	15.7 ± 1.2	6.77 ± 0.34	<1.6	151 ± 12
	Ground samples	37.6 ± 3.2	26.9 ± 3.4	26.9 ± 2.3	24.7 ± 1.9	26.0 ± 3.6	14.37 ± 0.56	15.28 ± 0.88	5.82 ± 0.33	<2.1	122.6 ± 4.8
	Calculated	34.2 ± 2.0	30.0 ± 3.7	30.8 ± 3.7	27.69 ± 0.81	29.2 ± 2.5	12.53 ± 0.36	14.58 ± 0.64	5.27 ± 0.17	1.15 ± 0.10	131.8 ± 6.4

The uncertainties cited are for a coverage factor k = 2.

**Table 11:** Activity concentrations (Bq kg^−1^) for: (a) hardened hybrid cement 5-cm cubic specimens; (b) ground mortars; and (c) values calculated from the percentage of individual components used to prepare mortars with granite sand.

Sample	Type	Uranium Series					Thorium Series			Actinium Series	^40^K
		^234^Th	^226^Ra	^214^Pb	^214^Pb	^210^Pb	^228^Ac	^212^Pb	^208^Tl	^235^U	
M1G-2d	Cubic specimens	158 ± 13	130 ± 13	153 ± 12	140 ± 14	153 ± 11	51.4 ± 3.4	56.2 ± 2.5	21.7 ± 1.0	6.4 ± 1.0	845 ± 63
	Ground samples	153 ± 12	130 ± 14	149.9 ± 8.1	137.8 ± 5.2	147 ± 15	48.8 ± 1.8	54.5 ± 3.1	21.4 ± 1.0	6.9 ± 1.2	892 ± 30
	Calculated	149 ± 10	129 ± 10	144 ± 10	134.1 ± 7.7	156 ± 15	45.3 ± 1.4	48.9 ± 2.4	20.02 ± 0.84	5.60 ± 0.65	741 ± 30
M1G-SP-2d	Cubic specimens	180 ± 10	138 ± 11	163.4 ± 6.2	147.9 ± 4.6	165 ± 13	54.3 ± 1.1	58.7 ± 2.4	23.25 ± 0.76	7.7 ± 1.0	902 ± 20
	Ground samples	160 ± 12	140 ± 13	155.0 ± 8.5	138.2 ± 3.1	160 ± 17	54.5 ± 1.7	56.8 ± 3.3	22.4 ± 1.0	7.4 ± 1.2	919 ± 28
	Calculated	153 ± 10	132 ± 11	148 ± 11	137.4 ± 7.9	160 ± 15	46.4 ± 1.5	50.1 ± 2.5	20.52 ± 0.86	5.74 ± 0.66	760 ± 30
M2G-2d	Cubic specimens	173 ± 17	137 ± 14	148.2 ± 7.8	134.4 ± 8.9	165 ± 12	52.1 ± 3.9	57.3 ± 3.3	22.3 ± 1.3	5.20 ± 0.85	814 ± 47
	Ground samples	159 ± 12	137 ± 12	147.8 ± 7.7	138.8 ± 6.1	148 ± 15	50.3 ± 2.8	53.9 ± 3.1	21.5 ± 1.0	6.20 ± 0.76	817 ± 25
	Calculated	154.5 ± 9.2	134 ± 10	150 ± 10	138.5 ± 7.3	156 ± 14	46.1 ± 1.4	50.1 ± 2.3	20.18 ± 0.80	5.94 ± 0.62	707 ± 28
M1G-28d	Cubic specimens	152.2 ± 8.1	123 ± 10	146.6 ± 5.5	135.1 ± 8.1	146 ± 11	50.3 ± 3.0	54.8 ± 2.7	21.6 ± 1.4	5.87 ± 0.88	840 ± 65
	Ground samples	147 ± 11	132 ± 12	146 ± 11	138 ± 13	152 ± 15	50.6 ± 3.4	51.8 ± 2.9	21.5 ± 1.3	6.8 ± 1.1	871 ± 36
	Calculated	149 ± 10	129 ± 10	144 ± 10	134.1 ± 7.7	156 ± 15	45.3 ± 1.4	48.9 ± 2.4	20.02 ± 0.84	5.60 ± 0.65	741 ± 30
M1G-SP-28d	Cubic specimens	172 ± 10	135 ± 10	155.5 ± 6.0	143.0 ± 2.2	160 ± 12	54.1 ± 1.1	58.4 ± 2.4	23.21 ± 0.77	8.19 ± 0.76	911 ± 20
	Ground samples	161 ± 12	139 ± 14	157.0 ± 8.5	145.1 ± 3.5	163 ± 17	54.8 ± 1.9	57.4 ± 3.4	22.6 ± 1.1	<5.6	955 ± 30
	Calculated	153 ± 10	132 ± 11	148 ± 11	137.4 ± 7.9	160 ± 15	46.4 ± 1.5	50.1 ± 2.5	20.52 ± 0.86	5.74 ± 0.66	760 ± 30
M2G-28d	Cubic specimens	161.0 ± 8.6	123.6 ± 8.8	143.0 ± 5.2	129.6 ± 5.6	127 ± 18	51.4 ± 2.4	56.4 ± 2.7	22.0 ± 1.2	7.3 ± 1.0	803 ± 35
	Ground samples	150 ± 11	125 ± 12	133.8 ± 7.1	123 ± 10	143 ± 15	47.8 ± 3.1	53.0 ± 3.0	21.1 ± 1.2	8.0 ± 1.8	803 ± 49
	Calculated	154.5 ± 9.2	134 ± 10	150 ± 10	138.5 ± 7.3	156 ± 14	46.1 ± 1.4	50.1 ± 2.3	20.18 ± 0.80	5.94 ± 0.62	707 ± 28

The uncertainties cited are for a coverage factor k = 2.

## Data Availability

The data presented in this study is available on request from the corresponding author.
